# Effectiveness of enhancing contact model on reducing stigma of mental illness among family caregivers of persons with schizophrenia in rural China: A cluster randomized controlled trial

**DOI:** 10.1016/j.lanwpc.2022.100419

**Published:** 2022-03-03

**Authors:** Mao-Sheng Ran, Yi-Zhou Wang, Pei-Yi Lu, Xue Weng, Tian-Ming Zhang, Shu-Yu Deng, Ming Li, Wei Luo, Irene Yin-Ling Wong, Lawrence H. Yang, Graham Thornicroft, Lin Lu

**Affiliations:** a Department of Social Work and Social Administration, University of Hong Kong, Hong Kong, China; b Mental Health Center, West China Hospital, Sichuan University, Chengdu, Sichuan 610041, China; c Department of Political Science, Iowa State University, United States; d Institute of Advanced Studies in Humanities and Social Sciences, Beijing Normal University at Zhuhai, Zhuhai, China; e Department of Social Work, Shanghai University, Shanghai, China; f Chengdu Xinjin Second People's Hospital, China; g School of Social Policy and Practice, University of Pennsylvania, United States; h Department of Social and Behavioral Sciences, New York University, United States; i Department of Epidemiology, Columbia University, United States; j Centre for Global Mental Health and Centre for Implementation Science, Institute of Psychiatry, Psychology and Neuroscience, King's College London, UK; k Peking University Sixth Hospital, Peking University Institute of Mental Health, Beijing, China

**Keywords:** Enhancing contact model, Intervention, Stigma, Family caregivers, China

## Abstract

**Background:**

Contact-based intervention has been documented and proved effective on reducing stigma of mental illness in high-income countries, but it is still unclear about the effectiveness of the contact-based intervention among family caregivers of persons with schizophrenia (FCPWS) in low- and middle-income countries including rural China.

**Methods:**

We conducted a cluster randomized controlled trial in FCPWS in eight rural townships in Xinjin district of Chengdu city in Southwest China. The FCPWS in these townships were randomly allocated to the Enhancing Contact Model (ECM), Psychoeducational Family Intervention (PFI), or Treatment as Usual (TAU) group. FCPWS in three groups were provided specific interventions and follow-ups. By using a mixed-effect model, our goal was to examine the differences in affiliate self-stigma scale (ASSS) scores among three groups with the data collected at baseline (T0), post-intervention (T1), 3-month (T2), and 9-month (T3) follow-up timepoints, respectively. This trial is registered with ChiCTR, number ChiCTR2000039133.

**Findings:**

In April 2019, 253 FCPWS from 8 townships were randomly assigned to receive either ECM (cluster=3, n=90), PFI (cluster=2, n=81), or TAU (cluster=3, n=82). Compared with participants in the TAU group, participants in the ECM group had statistically significantly lower ASSS scores at 9-month follow-up (estimated parameter [EP]= -5.51, 95% CI -10.27 to -0.74, p=0.02). There were no statistically significantly different ASSS scores at 9-month follow up between ECM and PFI groups. Compared with participants in the PFI group, younger (<60 years old), with higher monthly income and other caregiver (e.g., parent, sibling, child) participants in the ECM group had statistically significantly lower ASSS scores in the 3-month follow-up (EP = -5.66, 95% CI -10.13 to -1.19, p<0.01; EP = -7.82, 95% CI -11.87 to -3.78, p<0.001; EP = -6.79, 95% CI -10.69 to -2.90, p<0.001, respectively).

**Interpretation:**

This first trial in rural China shows that ECM intervention, a new anti-stigma intervention model, is a promising method for reducing affiliate stigma among FCPWS. The ECM intervention is more effective and stable than the PFI on reducing affiliate stigma among FCPWS. Further research needs to explore whether a long-term intervention could produce a more positive anti-stigma outcome trajectory.

**Funding:**

General Research Fund, University Grants Committee, Hong Kong SAR (GRF, Grant No. 17605618, 2018-2021, PI: Dr. M.S. Ran).

**Research in context**

**Evidence before this study**

We searched PubMed and CNKI (China National Knowledge Infrastructure) for articles in English and Chinese that were published up to April 1, 2021, with the keywords of “stigma”, “caregiver*”, “China”, “trial*”, “mental illness”, “schizophrenia”. One meta-analysis identified the correlates of affiliate stigma in family caregivers of persons diagnosed with schizophrenia and indicated the potential benefits of various interventions. However, we identified no trials of anti-stigma intervention for FCPWS in China. One systematic review identified 28 qualitative studies on the experience of family members caring for individual with early psychosis. Results highlighted the needs of initiating caregiver intervention to reduce the stigma-related burden among caregivers. Only one protocol showed that a cluster randomized controlled trial was underway to support family caregiving by using WeChat. However, no intervention was identified to focus on rural areas in Chinese culture background nor in any other developing communities. Lastly, the comparative effectiveness of different anti-stigma strategies (contact model vs family psychoeducation) is still unclear in China, not to mention the duration of effects and its implication in rural areas.

**Added value of this study**

To our knowledge, this is the first study to compare the effectiveness of the ECM, a new intervention model emphasizing positive contact, and the PFI on reducing affiliate stigma of FCPWS in rural China. Our study suggests that the ECM intervention is an acceptable, safe, and effective intervention for reducing affiliate stigma in FCPWS in rural China. Moreover, the ECM intervention is more effective and stable on reducing affiliate stigma of FCPWS than the PFI.

**Implications of all available evidence**

This study generates new knowledge of the ECM intervention for reducing affiliate stigma of FCPWS and extends existing knowledge of the contact model in reducing stigma of mental illness especially in rural China and other places with similar context as rural China in low- and middle-income countries. Our preliminary findings are important for facilitating the development of mental health policy and national evidence-based anti-stigma campaign on reducing stigma of mental illness, enhancing family caregiving quality and improving treatment and recovery of persons with schizophrenia.

Alt-text: Unlabelled box

## Introduction

The stigma of mental illness not only exerts adverse effects on persons with schizophrenia, but also has significant negative consequences on their family caregivers,^1^ such as affiliate stigma which refers to the prejudice and discrimination against those associated with persons with mental illness.^2^^,^^3^ For example, family members of persons with schizophrenia with affiliate stigma, may see stigmatization owing to their kinship with these patients.^2^ Affiliate stigma can have severe consequences on family caregivers of persons with schizophrenia (FCPWS), such as: (1) negatively influencing self-esteem, ability to keep friends, obtaining a job or place to live, and acceptance by others^2^^,^^4^; (2) minimizing help-seeking behavior and reducing the care quality^3^; and (3) encouraging negative coping strategies.^4^ Although FCPWS experience severe stigma, most previous anti-stigma programs to date have been conducted in high-income countries, but not low- and middle-income countries (e.g., China), and focused on reducing stigma in the general population rather than FCPWS.^5^^,^^6^

As most individuals with schizophrenia (over 90%) live with their family members who are their main caregivers in communities in many low- and middle-income countries including China, reducing the stigma of mental illness among family caregivers is critically important to promote treatment and recovery of persons with schizophrenia.^2^^,^^5^^,^^7^ Moreover, given the severe affiliate stigma in FCPWS, it is vital to develop effective anti-stigma interventions for them to reduce their stigma of mental illness, enhance the quality of family caregiving and improve community mental health care for persons with schizophrenia.^5^^,^^8^

The anti-stigma strategies adopted across the globe fall into three categories: protest or social activism, education, and intergroup contact.^5^^,^^6^^,^^8^^,^^9^ Although there is little empirical evidence supporting the efficacy of social activism, both education and contact have been found effective in reducing stigma. A meta-analysis of 79 programs representing 38,364 research participants from 14 countries concluded that contact was more effective than education in reducing stigma for adults.^10^ Combining knowledge and interpersonal contact constitutes an effective method to augment the educational effects of programs.^2^^,^^5^ A study of a peer-led psychoeducation caregiver program in Hong Kong, Taipei, and Bangkok demonstrated that contact provided by a peer-caregiver co-leader could enhance the effects of anti-stigma intervention.^12^ However, few studies have examined the long-term effectiveness of different anti-stigma interventions (e.g., contact and psychoeducational family intervention (PFI)) for family caregivers.^5^

Developed by Allport (1954), contact theory suggested that increasing social contact might decrease stigma, internalized stigma in particular, and discrimination.^11^^,^^12^ A growing body of research showed that positive and direct personal contact might be an effective anti-stigma strategy to promote acceptance.^5^^,^^13^ However, there is a dearth of randomized controlled trials (RCTs) exploring the effectiveness of contact based anti-stigma intervention for affiliate stigma of FCPWS in Chinese context, particularly in rural areas.^5^^,^^14^

Chengdu Mental Health Project (CMHP), starting in the early 1990s in rural China, is an ongoing longitudinal mental health project.^13^, ^14^, ^15^, ^16^ A mental health survey from the CMHP was conducted in Xinjin district (population: 152,776), Chengdu city in 2015, 671 persons with schizophrenia were identified and their family caregivers served as potential participants in this study. Our previous studies indicated that PFI was effective in improving treatment adherence and social functioning in persons with schizophrenia.^15^, ^16^, ^17^ However, the effectiveness of different anti-stigma strategies (e.g., contact model and psychoeducation) still remains unclear among FCPWS in rural China. Hence, the Enhancing Contact Model (ECM), a new model of comprehensive contact intervention emphasizing positive contact,^18^ was firstly proposed by Dr. Ran in 2018 and introduced in this study to test its effectiveness on reducing affiliate stigma of FCPWS. Positive contact was defined as equal, supportive, voluntary and pleasant contact.^18^, ^19^, ^20^ It was assumed that ECM intervention could reduce affiliate stigma by enhancing FCPWS positive contact (e.g., frequency and quality of contact) with persons with schizophrenia (individual contact) and other peer family caregivers of these patients (group contact).^18^

In this study we aimed to test whether the ECM intervention was more effective than either the PFI or treatment as usual (TAU) groups in reducing affiliate stigma in FCPWS in immediate (post-intervention), mid- (3-month follow-up) and long-term (9-month follow-up) follow-up in rural China.

## Methods

### Study design and setting

**Figure 1:**
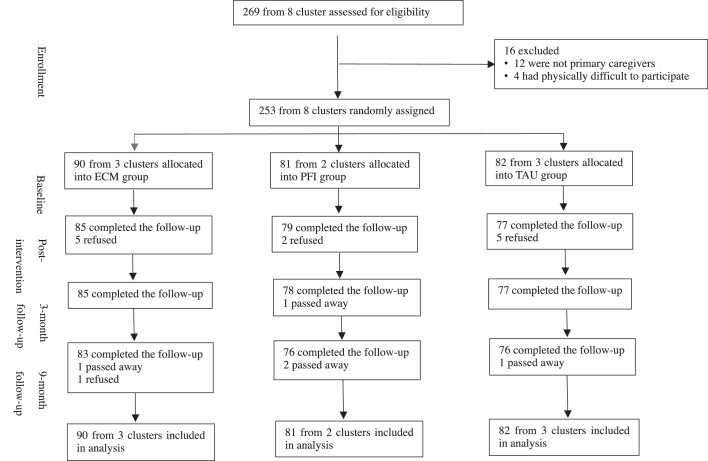
The CONSORT diagram of participation flow.

We conducted a parallel, three-arm, single-blinded, cluster randomized controlled trial in Xinjin district, Chengdu city in Southwest China (Figure 1). The trial was approved by the University of Hong Kong Human Research Ethics Committee (HKUHREC). The research protocol, approved by General Research Fund (GRF, Grant No: 17605618), University Grants Committee, Hong Kong.

### Participants

The inclusion criteria were: (1) being the main family caregivers of person diagnosed with schizophrenia by International Classification of Diseases 10th Revision; (2) aged 18–75 years old; and (3) living with and caring for persons with schizophrenia. The main family caregivers in this study were referring to these family members who take the major responsibility of caring persons with schizophrenia (e.g., time, effort, duty) in household. The exclusion criteria were: (1) likely to engage in an imminent risk behavior (e.g., suicide or violence); and (2) identified by a trained health professional as unsuitable to join the study (e.g., unable to communicate). Participants were referred to the study by the local mental health professionals (e.g., psychiatrists, primary health care providers) who were taking charge of patients’ regular community care management. Written informed consents were obtained from all participants, who were provided with a detailed explanation of the study's objectives, risks and benefits, the voluntary nature of participation, and their rights to withdraw. All family caregivers received around 140 RMB as compensation for their participation in all 12 sessions (e.g., around 12 RMB for each session).

### Randomization and masking

To reduce possible contamination between FCPWS in the same village, this study used a cluster randomized controlled trial. Eight rural townships in the Xinjin district, used as a cluster, served as the unit of randomization. In general, FCPWS in different townships live in different villages and are under different twonship governments and health care institutes (e.g., township hospital, village clinic). Randomization was conducted by a staff member of the local mental hospital who was not involved in the study, with a pre-determined list generated by an online randomization program (www.randomization.com) in the allocation sequence of 1:1:1 ratio. 3 townships were assigned to the Enhancing Contact Model intervention (ECM) group, 2 townships were assigned to the Psychoeducational Family Intervention (PFI) group, and 3 townships were assigned to the Treatment as Usual (TAU) group. FCPWS in these townships were allocated to the three groups accordingly. In view of the nature of the interventions, the trained research team member who delivered the intervention was aware of the research allocation, but all the local mental health professionals, assessors nor participants were blinded to it. Independent and well-trained assessors (e.g., undergraduate, psychological counsellor) were blind to the research design and completed the baseline and the follow-up assessments.

### Procedures

Participants who were allocated to receive the ECM intervention were offered a 12-session peer group on a weekly basis (approximately 90 min per session). Mixed with different delivering methods (e.g., training, discussion, role play, and take-home practice), the ECM intervention comprised two parts: (1) provision of brief psychoeducational training to improve the understanding of schizophrenia, psychiatric symptoms, treatment, and recovery (4 weeks)^7^^,^^15^^,^^16^; and (2) stigma-reducing via enhanced contact: (a) to increase single family contact with persons with schizophrenia (e.g., contact between family caregivers and persons with schizophrenia at home and in public) (4 weeks), and (b) to improve group family contact with other FCPWS (e.g., contact and support among peer FCPWS) in the community (4 weeks). This part emphasized frequency (e.g., times of contact) and quality of contact (e.g., positive contact) between persons with schizophrenia and FCPWS. The major contents in the ECM intervention are shown in Appendix 1. Each group included around 10 family caregivers (ranging from 8 to 15 participants). Via peer group support and sharing experiences, participants were introduced with how to cope with discriminatory experiences modeled on the behavioral problem-solving component. Real-life examples of successful adaptation to discrimination in the rural community in our previous studies were provided as well. Family member co-leaders were trained to facilitate a sense of reality and intimacy. To simulate an on-site real-life caring situation, 1 or 2 persons with schizophrenia serving as teaching participants, were invited to attend each session. Moreover, except the ECM intervention sessions, all participants in the ECM group were assigned take-home practice (e.g., positive contact) at home or community each week.

Similar to part 1 of the ECM intervention, the PFI focused on psychoeducation of the causes and effective treatment of schizophrenia, emphasizing the possibility of gradual stabilization and recovery.^8^^,^^17^ We also integrated concepts such as family beliefs, attitudes, stigma, medication, and treatment compliance. Building on the PFI approach,^7^^,^^15^, ^16^, ^17^ the PFI reflected a contemporary understanding of schizophrenia from bio-psycho-social perspectives, but focused on education and information rather than explicitly addressing stigma as in the ECM group. Serving as control, participants in the TAU group were visited at home or township hospitals by the trained primary mental health professionals (around 15 min each time) to discuss their general concerns about their relatives with schizophrenia at 4 timepoints respectively. Although these participants at the TAU group might seek treatment help on their own, we provided no further intervention. The ECM, PFI and TAU protocols developed by the research team were used to guide the intervention sessions and assessments.^7^^,^^16^

Based on the requirement of intervention provider, a researcher (with a Master degree in Psychology and 4-year counselling experience) was selected and accepted 3-day (around 30 h in total) intensive training program provided by the research team. The training program mainly included knowledge of schizophrenia and anti-stigma intervention (e.g., ECM and PFI). The intervention provider was assessed by research team after the training program and met the requirement for delivering both the ECM and the PFI interventions. Moreover, the trained intervention provider attended weekly group supervision sessions with the research team at University of Hong Kong (HKU) via Skype or WeChat and bimonthly field supervision in the Xinjin district, Chengdu city. A trial steering committee was established to oversee the activities in all three groups. There was no harm assessed in the study.

### Measurements

Assessments took place in participants’ homes, villages, clinics or township hospitals. The measures included: (1) demographic characteristics of persons with schizophrenia and FCPWS; (2) intervention adherence of FCPWS in the ECM group; and (3) the Affiliate Self-Stigma Scale (ASSS) of FCPWS. The intervention adherence of FCPWS was measured according to their adherence to the take-home practice (positive contact with persons with schizophrenia). The ASSS was administered to measure family caregivers’ changes in their affiliate stigma.^19^, ^20^, ^21^, ^22^, ^23^ The ASSS, including 22 items, was developed into three dimensions: cognitive (7 items), affective (7 items) and behavioral (8 items).^21^ For example, the ASSS items include “Others will discriminate against me if I am with my family member with mental illness”, “I feel inferior because one of my family members has mental illness”, and “I dare not tell others that my family member has mental illness”. The Chinese version of the ASSS, measured on a 4-point Likert scale (1 = strongly disagree; 4 = strongly agree) has demonstrated good internal consistency (Cronbach's α = .94).^20^^,^^21^ Higher scores indicate higher level of affiliate stigma.

### Sample size

With reference to other anti-stigma intervention studies, this study (with a 3-arm, 4-time point design) was expected to have a moderate effect size (0.2).^6^ Assuming 90% power, a significance criterion of 0.05, and 0.5 as the correlation among repeated measures (Multivariate Analysis of Variance, repeated measures, between factors),^24^ 201 family caregivers were needed, as calculated by the statistical software G-Power 3.1.9.2.^6^^,^^22^ Despite between-cluster variation may decrease power,^25^ we did not account design effect due to the budget constraint and the pragmatic nature of the trial. Assuming an attrition rate of 15%, 231 family caregivers from 8 clusters were needed in total for three arms (e.g., 77 in each arm). There was no allowance for multiplicity in the sample size calculation.

### Statistical analysis

We included all participants who were enrolled in the study in the analyses, including those who dropped out or were lost to follow-up (intention to treat). Accroding to the statistical analysis plan, the analysis of variance and Pearson chi-square statistic were used for continuous and categorical variables respectively. We calculated an intra-cluster correlation coefficient (ICC) to assess the proportion of variance in study outcomes. We further used a mixed-effect model with unstructured covariance structure for continuous variables with four repeated measures at baseline (T0), post-intervention (T1), 3-month follow-up (T2), and 9-month follow-up (T3). We calculated standard errors and confidence intervals using robust estimation methods to account for the clustering of observations. By controlling for family caregivers’ education, sex, age, marital status, and household income in the mixed-effect model,^24^^,^^26^, ^27^, ^28^ we estimated the mean ASSS score among the three groups at the immediate (T1), mid-term (T2) and long-term (T3). The Kenward‐Roger approximation was used to estimate denominator degrees of freedom. We took the cluster effect into consideration by the following. First, we randomized the cluster as unit rather than as individual to ensure the potential cluster effect was generated by chance. Moreover, we made a two levels of nesting constructure in the model: Level 1 is the nesting of different timepoints within each individual, and Level 2 is within the township, the clusters. The present model included random effects of intercept (baseline ASSS score) and slope (time: baseline, post-intervention, 3-month and 9-month follow-ups), one fixed effect (group: ECM, PFI, and TAU), and the Group × Time interaction. Additionally, sensitivity analysis was conducted to examine if treating time as an ordinal or continuous variable and/or using linear or quadratic growth models altered the results. Findings suggested none of them changed the key findings (see Appendix 2).

In order to induce a robust statistical reference, we adjusted the pre-specified subgroups to achieve the balance of sample size in different groups. The adjusted subgroup analyses examined whether intervention effects differed by age group, sex, personal monthly income and relationship. After adjusting for the baseline covariates, linear regression method was used to compare the variable “positive contact” and “positive contact sites” respectively, and Pearson association between “times of positive contact” and the variables of interest. Missing data were assumed missing at random (MAR). First, multiple imputation by fully conditional specification was used to address missing data. Second, the 5 imputed datasets were then analyzed using a mixed effect model with both fixed and random effect. Third, the coefficient estimates (e.g., treatment difference) obtained from each analyzed dataset were then pooled for inference. Data analysis engaged 3 researchers and was processed and verified by using SAS software, version 9.4.

### Role of the funding source

The funder of the study had no role in study design, data collection, data analysis, data interpretation, or writing of the report. The corresponding author had full access to all the data in the study and had final responsibility for the decision to submit for publication.

## Results

We randomized 8 rural townships with FCPWS sample size ranging from 17 to 55. The ICC for primary outcome was 0.04 before the imputation and 0.07 after the imputation. These townships belong to the Xinjin district, Chengdu city, Sichuan province, which has an average gross domestic product (GDP) among all the provinces of China. In April 2019, among 269 FCPWS, 253 were eligible and consented to participate. 90 of them were randomized at the township level to the ECM group, 81 to the PFI group and 82 to the TAU group (see Figure 1). The average attendance rates of FCPWS were above 95% in both the ECM and the PFI groups each session. Our overall retention rate was above 92% and these were similar across groups at all follow-ups. Baseline characteristics of FCPWS were summarized in Table 1. There was no statistically significant difference among three groups in the demographic features at baseline.

**Table 1:** Demographic characteristics of participants at baseline assessment (n=253).

	ECM (n=90)	PFI (n=81)	TAU (n=82)
	N (%)	N (%)	N (%)
Number of clusters	3	2	3
Mean cluster size	30	41	27
Sex			
Male	48 (53.33)	42 (51.85)	43 (52.44)
Female	42 (46.67)	39 (48.15)	39 (47.56)
Marital status:			
Single	4 (4.44)	2 (2.47)	2 (2.44)
Married	78 (86.67)	69 (85.19)	64 (78.05)
Divorced	2 (2.22)	2 (2.47)	3 (3.66)
Widowed	5 (5.56)	7 (8.64)	13 (15.85)
Others (e.g., remarried)	1 (1.11)	1 (1.23)	0 (0)
Employment			
With a full-time paid job	68 (76.56)	44 (54.32)	51 (62.20)
With a part-time paid job	5 (5.56)	6 (7.41)	6 (7.32)
Without a paid job	17 (18.89)	31 (38.27)	25 (30.49)
With family members who are working outside of Xinjin			
Yes	23 (25.56)	25 (30.86)	23 (28.05)
No	67 (74.44)	56 (69.14)	59 (71.95)
Relationship with caregivers:			
Parents	26 (28.89)	27 (33.33)	31 (37.80)
Spouse	43 (47.78)	34 (41.98)	37 (45.12)
Siblings	5 (5.56)	6 (7.41)	5 (6.10)
Children	12 (13.33)	9 (11.11)	8 (9.76)
Others (e.g., uncles, aunts)	4 (4.44)	5 (6.17)	1 (1.22)
	Mean/Median (SD)		
Age (years)	59.8 (12.9)	60.8 (13.2)	60.7 (13.6)
Education (years)	6 (6 to 9)	6 (6 to 9)	6 (5.5 to 9)
Household annual income (RMB)	14700 (8400 to 27600)	24000 (12000 to 40920)	20400 (10000 to 36000)
Number of family members	3 (3 to 5)	3 (3 to 5)	3 (2 to 4)
Baseline of ASSS score, SE	50.58 (1.68)	50.16 (1.19)	54.01 (1.53)

Note: ECM = enhancing contact model; PFI = psychoeducational family intervention; TAU = treatment as usual. The participants of three arms from 8 clusters (townships). Education, household annual income and number of family members are medians (interquartile range); age is mean (standard deviation).

Table 2 shows participants’ outcomes of the overall and sub-domain ASSS scores. There were no statistically significant differences of ASSS scores at baseline assessment among three groups. The observed effect is consistent with our hypothesis that the ECM was more effective than the control in reducing family caregivers’ self-stigma; while it is inconsistent with our hypothesis that the ECM was more effective than the PFI in reducing family caregivers’ self-stigma. At the post-intervention (T1), participants in the ECM group had statistically significantly lower total ASSS scores than those in the TAU group (Estimated Parameter (EP) = -4.29, 95% CI -7.98 to -0.61, p=0.02). There were no statistically significant differences of total ASSS scores between the ECM and the PFI groups. For the affective domain, participants in the ECM group had statistically significantly lower ASSS scores than those in the TAU group (EP = -1.62, 95% CI -2.97 to -0.28, p=0.02), while participants in the ECM group had no statistically significant differences of ASSS scores than those in the PFI group. As for the behavioral domain, we did not find statistically significant differences of ASSS scores among three groups. As for the cognitive domain, participants in the ECM group had statistically significantly lower ASSS scores than those in the TAU group (EP = -1.46, 95% CI -2.75 to -0.17, p=0.03), while participants in the ECM group had no statistically significant differences of ASSS scores than those in the PFI group.

**Table 2:** Participants’ outcomes of the overall and sub-domain ASSS scores (intention-to-treatment analysis).

	Estimated in ECM (N=90) (Mean, SE)	Estimated in PFI (N=81) (Mean, SE)	Estimated in TAU (N=82) (Mean, SE)	Treatment Difference			
				ECM vs PFI (EP, 95% CI)	P value	ECM vs TAU (EP, 95% CI)	P value
**Primary outcome:**							
**Stigma:**							
**post-intervention (T1)**	45.58 (1.32)	44.99 (1.36)	49.93 (1.41)	0.20 (-3.57 to 3.97)	0.9158	-4.29 (-7.98 to -0.61)	0.0224
**3-month follow-up (T2)**	45.11 (1.55)	48.52 (1.68)	46.65 (1.78)	-3.80 (-8.37 to 0.78)	0.1041	-1.49 (-6.15 to 3.17)	0.5311
**9-month follow-up (T3)**	47.63 (1.75)	46.98 (1.83)	53.19 (1.68)	0.26 (-4.60 to 5.12)	0.9155	-5.51 (-10.27 to -0.74)	0.0235
**Secondary outcomes:**							
**Stigma-Affective:**							
**baseline (T0)**	17.79 (0.62)	17.25 (0.45)	19.00 (0.58)	-0.05 (-1.44 to			
**post-intervention (T1)**	15.73 (0.46)	15.73 (0.50)	17.43 (0.52)	1.34)	0.9415	-1.62 (-2.97 to -0.28)	0.0181
**3-month follow-up (T2)**	15.34 (0.51)	16.56 (0.56)	15.93 (0.61)	-1.28 (-2.81 to 0.25)	0.1015	-0.58 (-2.14 to 0.98)	0.4671
**9-month follow-up (T3)**	16.54 (0.60)	16.54 (0.64)	18.41 (0.58)	-0.03 (-1.68 to 1.62)	0.9709	-1.80 (-3.44 to -0.16)	0.0319
**Stigma-Behavioral:**							
**baseline (T0)**	16.88 (0.58)	16.73 (0.46)	17.68 (0.50)				
**post-intervention (T1)**	15.24 (0.46)	14.94 (0.49)	16.43 (0.46)	0.15 (-1.24 to 1.54)	0.8248	-1.19 (-2.50 to 0.13)	0.0765
**3-month follow-up (T2)**	15.68 (0.59)	16.59 (0.61)	15.90 (0.63)	-1.08 (-2.86 to 0.69)	0.2278	-0.04 (-1.67 to 1.58)	0.9596
**9-month follow-up (T3)**	15.79 (0.63)	15.72 (0.68)	17.58 (0.64)	-0.26 (-2.11 to 1.60)	0.7862	-1.85 (-3.64 to -0.07)	0.0420
**Stigma-Cognitive:**							
**baseline (T0)**	15.91 (0.58)	16.19 (0.43)	17.33 (0.58)				
**post-intervention (T1)**	14.60 (0.48)	14.34 (0.45)	16.06 (0.51)	0.09 (-1.23 to 1.42)	0.8903	-1.46 (-2.75 to -0.17)	0.0266
**3-month follow-up (T2)**	14.09 (0.54)	15.38 (0.57)	14.82 (0.61)	-1.45 (-3.01 to 0.12)	0.0699	-0.74 (-2.33 to 0.86)	0.3653
**9-month follow-up (T3)**	15.30 (0.61)	14.73 (0.61)	17.20 (0.57)	0.45 (-1.21 to 2.11)	0.5954	-1.85 (-3.47 to -0.23)	0.0251

Note: The analysis based on the intention-to-treatment population (N=253). Treatment difference analysis was based on linear mixed-effect model after adjusting for baseline demographic characteristics; SE=Standard Error; EP=Estimated Parameter; CI=Confidential Interval.

At 9-month follow-up (T3), participants in the ECM group had statistically significantly lower total ASSS scores than those in the TAU group (EP = -5.51, 95% CI -10.27 to -0.74, p=0.02). For the affective, behavioral and cognitive domains, participants in the ECM group had statistically significantly lower ASSS scores than those in the TAU group (EP = -1.80, 95% CI -3.44 to -0.16, p=0.03; EP = -1.85, 95% CI -3.64 to -0.07, p=0.04; EP = -1.85, 95% CI -3.47 to -0.23, p=0.03; respectively), while participants in the ECM group had no statistically significant differences of ASSS scores compared with those in the PFI group.

**Figure 2:**
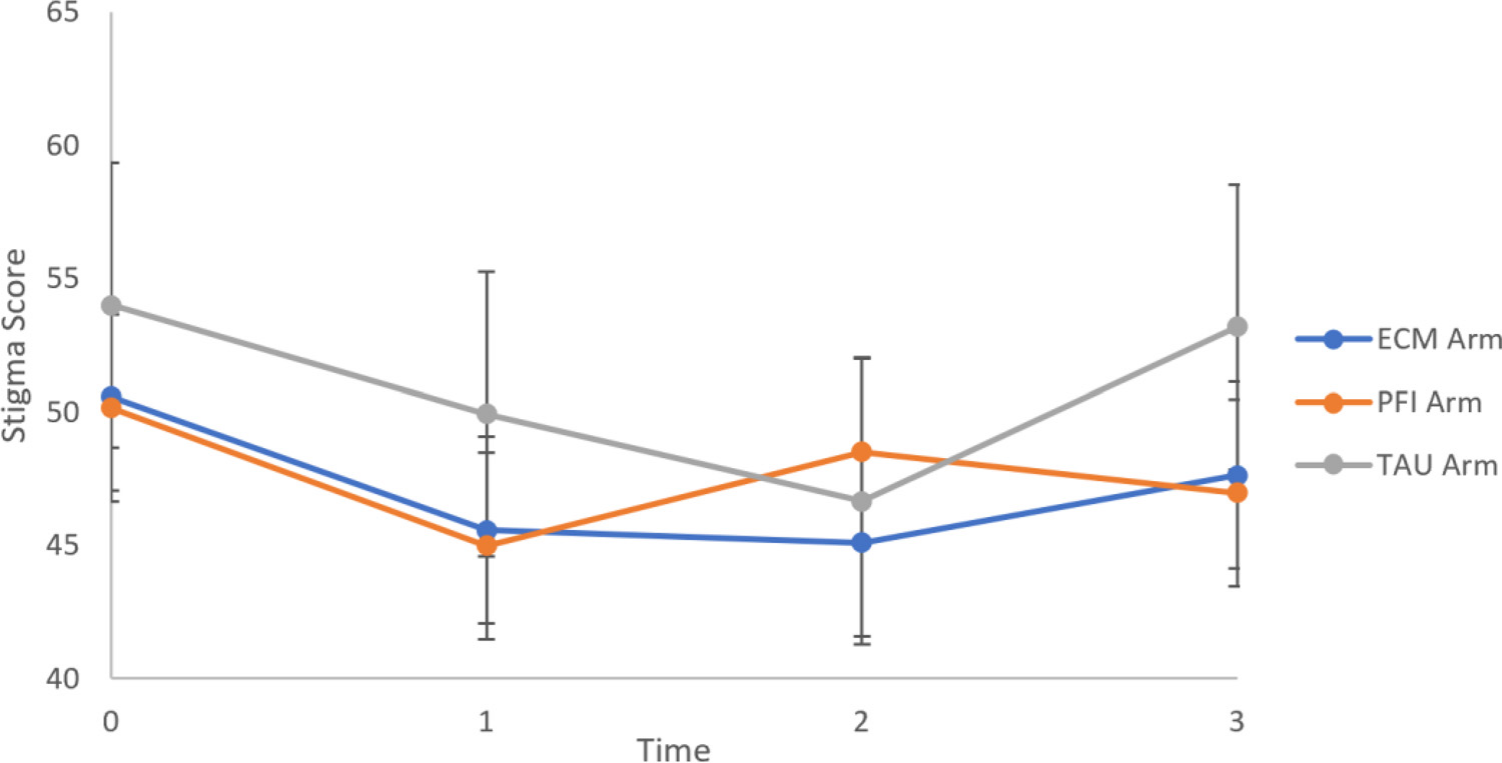
Predicted family caregivers’ affiliate stigma outcome trajectory over time Note: Predicted stigma outcome was computed based on linear mixed-effect model after adjusting for baseline demographic characteristics. Time: 0 = baseline, 1 = post-intervention, 2 = 3-month follow-up, 3 = 9-month follow-up; ECM = enhancing contact model; PFI = psychoeducational family intervention; TAU = treatment as usual; The high and low lines are 95% CI.

Figure 2 illustrates estimated participants’ affiliate stigma outcomes trajectory over time based on mixed-effect model. Different patterns of participants’ affiliate stigma for three groups were observed. As for the ECM group, there had been a steep fall in the total ASSS scores since the intervention and reached the lowest point at 3-month follow-up, and then a slightly rise occurred at 9-month follow-up. As for the PFI group, there had been a sharp drop at post-intervention, with a rise at 3-month follow-up, and followed by a decrease at 9-month follow-up. As for the TAU group, there was a similar pattern with the ECM group, with a drop firstly before 3-month follow-up and then an increase at 9-month follow-up. However, the differences of the total ASSS scores were statistically significant between the ECM and the TAU groups at post-intervention and 9-month follow-up. What stands out in Figure 2 is the variability of groups at 3-month follow-up: the mean total score for the ECM and the TAU groups remained with a relatively falling trend, which was consistent with pre-test and post-intervention, and hit the lowest peak during the course. However, the mean score for the PFI group at 3-month follow-up was in a rise which was inconsistent with pre-test and post-intervention.

Table 3 shows the results of subgroups analysis of 3-month follow-up (T2) on ASSS scores. There were statistically significantly lower ASSS scores in younger participants (< 60 years old) in the ECM group than those in the PFI group (EP = -5.66, 95% CI -10.13 to -1.19, p<0.01), and there were statistically significantly lower ASSS scores in younger participants (<60 years old) in the PFI group than those in the TAU group (EP = 4.96, 95% CI 0.41 to 9.51, p=0.02). There were statistically significantly lower ASSS scores in female participants in the ECM group than those in the TAU group (EP = -4.79, 95% CI -8.99 to -0.60, p=0.01). Male participants in the PFI group had statistically significantly higher ASSS score than those in the TAU group (EP = 4.60, 95% CI 0.51 to 8.69, p=0.02). In participants with higher monthly income (RMB), the ASSS scores were statistically significantly lower in the ECM group than those in the PFI group (EP = -7.82, 95% CI -11.87 to -3.78, p<0.001). The ASSS scores in participants with higher monthly income (RMB) were statistically significantly higher in the PFI group than those in the TAU group (EP = 4.99, 95% CI 0.95 to 9.04, p=0.01). In other caregivers (e.g., parent, sibling, child), the ASSS scores were statistically significantly lower in the ECM group than those in the PFI group (EP = -6.79, 95% CI -10.69 to -2.90, p<0.001).

**Table 3:** The results of subgroup analysis of ASSS scores in 3-month follow-up (intention-to-treatment analysis).

	Estimated in ECM N, EP (95% CI)	Estimated in PFI N, EP (95% CI)	Estimated in TAU N, EP (95% CI)	P value for the Interaction	Treatment Effect (95%CI)					
					ECM vs PFI (EP, 95% CI)	P value	ECM vs TAU (EP, 95% CI)	P value	PFI vs TAU (EP, 95% CI)	P value
Age group (years)				0.0309						
**< 60**	41, 44.54(42.50 to 46.57)	32, 50.20(47.89 to 52.50)	38, 45.24(43.12 to 47.35)		-5.66(-10.13 to -1.19)	0.0042	-0.70 (-4.97 to 3.57)	0.9972	4.96(0.41 to 9.51)	0.0232
≥60	49, 45.58(43.72 to 47.44)	49, 47.42(45.56 to 49.28)	44, 47.88(45.92 to 49.84)		-1.84(-5.67 to 1.99)	0.7455	-2.30(-6.23 to 1.64)	0.5547	-0.46(-4.40 to 3.48)	0.9995
Sex				0.0035						
Male	48, 45.09(43.21 to 46.96)	42, 48.30(46.30 to 50.30)	43, 43.70(41.72 to 45.68)		-3.21(-7.19 to 0.78)	0.1962	1.39(-2.57 to 5.36)	0.9169	4.60(0.51 to 8.69)	0.0172
Female	42, 45.12(43.12 to 47.12)	39, 48.75(46.67 to 50.83)	39, 49.92(47.84 to 51.92)		-3.63(-7.83 to 0.57)	0.1344	-4.79(-8.99 to -0.60)	0.0145	-1.16(-5.44 to 3.11)	0.9714
Personal monthly income (RMB)				0.0001						
< 5**0**0	48, 47.17(45.30 to 49.04)	36, 45.94(43.79 to 48.10)	40, 47.78(45.73 to 49.83)		1.22(-2.93 to 5.38)	0.9600	-0.61(-4.65 to 3.42)	0.9980	-1.84(-6.17 to 2.49)	0.8315
≥5**0**0	42, 42.75(40.76 to 44.75)	45, 50.57(48.64 to 52.50)	42, 45.58(43.58 to 47.58)		-7.82(-11.87 to -3.78)	0.0001	-2.83(-6.94 to 1.28)	0.3628	4.99(0.95 to 9.04)	0.0058
Relationship				0.0012						
Spouse	43, 47.42(45.44 to 49.40)	34, 46.76(44.54 to 48.99)	37, 46.68(44.54 to 48.81)		0.65(-3.68 to 4.99)	0.9981	0.74(-3.50 to 4.98)	0.9962	0.09(-4.40 to 4.58)	1.000
Others (e.g., parent, sibling, child)	47, 42.99(41.09 to 44.88)	47, 49.78(47.89 to 51.68)	45, 46.64(44.70 to 48.57)		-6.79(-10.69 to -2.90)	0.0001	-3.65(-7.59 to 0.29)	0.0883	3.15(-0.80 to 7.09)	0.20370

Note: The analysis based on the intention-to-treatment population (N=253). ASSS scores in 3-month follow-up were based on linear regression analysis including intervention, subgroup and the interaction between intervention and subgroup. EP=Estimated Parameter.

Table 4 shows the intervention adherence of FCPWS’ take-home practice (e.g., positive contact) in the ECM group. We found that 94.3% participants in the ECM group used enhancing contact skills (e.g., positive contact) contacting with their mentally ill relatives at home and in public. Among them, 80.7% participants conducted positive contact at home, 6.0% in public, and 13.3% at home and in public. The mean time of positive contact per week between FCPWS and persons with schizophrenia was 5.68.

**Table 4:** The analysis of intervention adherence during the 9-month follow-up (n=253).

Adherence of take-home practice	Number (%)	Estimated Mean ASSS (95% CI)	P value
Positive contact (n=88)			0.63
Yes	83 (94.3)	46.44 (43.88 to 49.00)	
No	5 (5.7)	43.80 (33.37 to 54.23)	
Positive contact sites (n=83)			0.68
At home	67 (80.7)	46.04 (43.13 to 48.95)	
In public	5 (6.0)	50.90 (40.25 to 61.55)	
Both at home and in public	11 (13.3)	46.86 (39.69 to 54.04)	
	Mean (SD)		
Times of positive contact (per week)	5.68 (3.88)		

Note: The intervention: the take-home practice (e.g., positive contact).

## Discussion

To the best of our knowledge, this is the first randomized controlled trial in rural China to examine the effectiveness of the ECM intervention on reducing the affiliate stigma of FCPWS. Given the negative contact may increase stigma,^18^ the ECM intervention, a new intervention model, emphasizes positive contact with persons with schizophrenia. Allport's theory specified contact at equal power levels for stigma change to occur,^13^ but this type of contact with persons with schizophrenia may be even less likely to occur in rural China given the lack of peer supports and the limited treatment (e.g., antipsychotic medication) for persons with schizophrenia in this setting.^7^^,^^15^^,^^16^ Thus, the present study extends the contact theory (e.g., specifically indicating the role of positive contact) and sheds light on the effectiveness of the ECM intervention on reducing affiliate stigma in FCPWS in rural China. Furthermore, the final sample size (N=253) was larger than the sample size required (N=231) and the retention rate in this study was above 92% in total which is higher than most of its counterparts,^29^^,^^30^ suggesting a high data quality. Moreover, by doing the follow-up measurements, we examined the mid- (e.g., 3-month) and long-term (e.g., 9-month) effectiveness of different anti-stigma approaches (e.g., ECM and PFI) which provides evidence on the effect-maintenance period of the ECM intervention.

The results of this study show that the ECM intervention, combining education and positive contact, is an effective and stable method for reducing affiliate stigma of FCPWS in rural China. Firstly, compared with the TAU group, the ECM group had a stable performance with better anti-stigma outcome (e.g., reducing affiliate stigma) at immediate (post-intervention) and long-term (9-month follow-up), which is consistent with previous research findings.^31^, ^32^, ^33^, ^34^ On the other hand, there were no statistically significant differences of reducing stigma (e.g., total ASSS scores) between the ECM and the PFI groups at post-intervention, 3-month follow-up and 9-month follow-up, which also accords with earlier meta-analysis evidence.^10^ However, the effects of reducing stigma in the PFI group were temporary and fluctuating since the PFI group even had a higher ASSS score than the TAU group at 3-month follow-up (Jan 2020 when COVID-19 broke out). In subgroup analysis of ASSS scores in 3-month follow-up, participants in the ECM group (e.g., younger (<60 years old), other caregivers (e.g., parent, sibling, child) and with higher monthly income (≥500 RMB)) had statistically significantly lower ASSS scores than those in the PFI group (p<0.01). These results indicate that the ECM intervention is more effective and stable than the PFI on reducing affiliate stigma of FCPWS, especially for those younger, with higher monthly income and other family caregivers, which is consistent with previous studies indicating the potential effects of positive contact.^19^^,^^33^, ^34^, ^35^

This study may also suggest the potential benefit of the ECM intervention, such as less reliance on mental health professionals due to the characteristics of the ECM intervention, even during the period of COVID-19 epidemic with limited mental health resources.^36^ The results indicate that the ECM intervention may be especially suitable for areas with limited mental health resources (e.g., mental health institutes and professionals). However, further studies should be conducted to explore the mechanism of the effective ECM intervention, and different effects of the ECM intervention and the PFI on reducing affiliate stigma in FCPWS in different areas.

This study supports that the short and long-term (e.g., 9-month follow-up) effects of the ECM intervention on reducing affiliate stigma in FCPWS are strong in rural China, a non-Western country.^37^ It implies a promising effectiveness of the ECM intervention on reducing affiliate stigma of FCPWS in other low- and middle-income countries with a similar context with rural China (e.g., most persons with schizophrenia are cared for by their family caregivers at home or community). Additionally, the results of this study indicate that both psychoeducation and contact-based strategies are effective on reducing affiliate stigma, even though the ECM intervention may be more stable and effective than the PFI.^10^ It is crucial to combine both psychoeducation and contact-based strategies on reducing stigma of mental illness in further mental health treatment and intervention.

Compared with the TAU group, participants in the ECM group had statistically significantly lower ASSS scores in the affective, behavioral and cognitive domains at 9-month follow-up, which is consistent with previous observation.^3^ The findings indicate that the ECM intervention may have different effects on affective, behavioral and cognitive domains of ASSS scores. Further studies should be conducted to identify the effectiveness of the ECM intervention on various domains of stigma of mental illness. Importantly, specific anti-stigma interventions focusing on different domains of stigma of mental illness should be further developed.^3^

This is the first study to explore the intervention adherence of contact model intervention. The results of this study showed that most FCPWS (94.3%) in the ECM group followed take-home practice to use positive contact skill during 3-month and 9-month follow-up, which is much higher than the average rate of adherence (67%) to mental health clinical practice among other trials.^38^ The high intervention adherence in this study indicates: (1) the ECM intervention is acceptable and fitting for FCPWS in rural China; (2) the quality of findings of this follow-up study is relatively high; and (3) FCPWS in rural China need community mental health services or intervention to improve their family care and facilitate mental health recovery for their relatives with mental illness. Authors of this study also suggest that the intervention adherence should be included as an important assessment aspect for improving the quality of anti-stigma interventions.

This study has several limitations. First, we did not take into account ICC in the sample size calculation and we might not effectively compare the different effects between the PFI and the TAU groups. Nevertheless, our traial is one of the largest trials compared to prior studies on FCPWS.^5^^,^^16^ Further fully powered trials are warranted to test the true effect between the ECM group and the PFI and the TAU groups. Second, because of the diversity in the participants’ socioeconomic characteristics in rural China, our findings may not be generalizable to developed countries or urban areas. However, our sample site, Xinjin district, Chengdu city, has an approximately median level GDP per capita in China, the findings of this study may be generalized to other areas with similar socioeconomic status. Third, the vulnerability of FCPWS might be impacted by the outbreak of COVID-19 during the study period (e.g., from January to September 2020), even though the results of this study might not be impacted severely. Further studies should be conducted to explore the potential impact of the COVID-19 epidemic. Moreover, all authors of this study suggest that it is important to investigate whether a long-term intervention approach could extend more positive outcome trajectories (e.g., extra maintenance session in the 3-month follow-up). The impact of culturally specific values (e.g., filial piety, face concern) should also be examined in further intervention studies.

Although community mental health care has been developed in current China, over 90% of persons with schizophrenia are cared for by their family caregivers at home in rural areas.^15^^,^^16^^,^^18^ Given the limited community mental health services and the important role of family caregivers in caring for persons with schizophrenia in China, effective anti-stigma interventions for these caregivers need to be developed. This study tested the effectiveness of the ECM intervention, a new anti-stigma model, on reducing affiliate stigma of FCPWS which contributes to the contact theory by emphasizing the specific role of positive contact. Reducing affiliate stigma of family caregivers should be crucial for strengthening their self-esteem and hope, enhancing their family caregiving quality, improving patients’ early treatment and long-term outcome, and facilitating their reintegration into the society.^6^^,^^11^^,^^20^ Beyond further testing of this anti-stigma model in other rural and urban areas, specific mental health policy on reducing stigma of mental illness and national evidence-based anti-stigma campaign should be developed in China to facilitate various anti-stigma interventions and improve mental health services for persons with schizophrenia and their family caregivers.

### Data sharing statement

The de-identified data are available on reasonable request to the corresponding author.

### Editor note

The Lancet Group takes a neutral position with respect to territorial claims in published maps and institutional affiliations.

### Contributors

MSR designed this study. MSR, YZW, WL and ML conducted this study. MSR, YZW, TMZ, YIW, ML and WL collected data. YZW, PYL, XW and SYD conducted data analysis. YZW and MSR wrote the first draft of the paper. All authors made contributions to critical revision of the manuscript.

## Declaration of interests

The authors declare no conflict of interest.

## Appendix Supplementary materials

Image, application 1

Image, application 2
