# Supplementary material for: Effectiveness of enhancing contact model on reducing stigma of mental illness among family caregivers of persons with schizophrenia in rural China: A cluster randomized controlled trial
Source: Lancet Reg Health West Pac. 2022 Mar 3;22:100419. doi: 10.1016/j.lanwpc.2022.100419 (PMC8897707; doi:10.1016/j.lanwpc.2022.100419)
Supplement: Supplementary file 1 [file mmc1.docx]

**Appendix 1. Sessions of the ECM intervention**

| No. of sessions | Theme | Major content |
| --- | --- | --- |
| ***Psychoeducation sessions*** | | |
| 1 | Schizophrenia | Introduction of schizophrenia, treatment, relapse, and family and community care |
| 2 | Caring and Burdens | Antipsychotic medication, psychological intervention, and family caring burden |
| 3 | Treatment, outcome and stigma of mental illness | Treatment, intervention and outcome of schizophrenia, and stigma of mental illness |
| 4 | How to reduce stigma of mental illness | Affiliate stigma of family caregivers, and how to reduce affiliate stigma and discrimination of family caregivers |
| ***Enhancing Contact Model (ECM) sessions*** | | |
| ***A. ECM (single family intervention)*** | | |
| 5 | To establish peer support group and accept patients | How to recognize and accept the persons with mental illness, how to establish the peer support group, and how to enhance positive contact |
| 6 | To facilitate mutual supportive relationship between patients and family caregivers | How to establish the mutual supportive relationship, how to solve the potential problems in mutual contact, how to improve the family care, and sharing and practice: positive contact |
| 7 | To understand positive contact and support | How to share experience of improving positive contact and communication, how to facilitate the positive supportive relationship, and sharing and practice: positive contact |
| 8 | To enhance positive contact and understanding | How to facilitate peer support and communication, how to enhance positive contact, understanding and support, and sharing and practice: positive contact |
| ***B. ECM (group family intervention)*** | | |
| 9 | To share experience of positive contact and facilitate communication | How to facilitate positive contact and to enhance contact behaviour skills; How to solve the potential problems in the contact and communication, and group sharing and practice: positive contact |
| 10 | To improve family care and community care | How to understand patients’ mental illness, the importance of treatment and recovery, how to facilitate family caregivers’ quality of caregiving, and group sharing and practice: positive contact |
| 11 | To reduce stigma, improve hope and quality of life | How to reduce stigma of mental illness in family caregivers, understand the prejudice and discrimination, facilitate mutual understanding and support, improve caregivers’ hope and quality of life, and group sharing and practice: positive contact |
| 12 | To reduce stigma and protect human rights of patients and their families | How to reduce stigma of mental illness, facilitate reintegration of persons with schizophrenia into society, and protect human rights of these patients and their family caregivers, group sharing and practice: positive contact |
