# Supplementary material for: Effectiveness of enhancing contact model on reducing stigma of mental illness among family caregivers of persons with schizophrenia in rural China: A cluster randomized controlled trial
Source: Lancet Reg Health West Pac. 2022 Mar 3;22:100419. doi: 10.1016/j.lanwpc.2022.100419 (PMC8897707; doi:10.1016/j.lanwpc.2022.100419)
Supplement: Supplementary file 2 [file mmc2.docx]

**Appendix 2. Sensitivity test**

|  | Linear model with “time” as an ordinal variable | Quadratic model with “time” as an ordinal variable | Linear model with “time” as a continuous variable | Quadratic model with “time” as a continuous variable |
| --- | --- | --- | --- | --- |
| 2LogLike | 8095.8 | 8087.8 | 8160.3 | 8135.0 |
| AIC (Akaike information criterion) | 8111.8 | 8103.8 | 8176.3 | 8151.0 |
| BIC (Bayesian information criterion) | 8140.1 | 8132.1 | 8204.6 | 8179.2 |
| CAIC (Consistent Akaike information criterion) | 8148.1 | 8140.1 | 8212.6 | 8187.2 |
